# Genomic Markers for Essential Tremor

**DOI:** 10.3390/ph14060516

**Published:** 2021-05-27

**Authors:** Félix Javier Jiménez-Jiménez, Hortensia Alonso-Navarro, Elena García-Martín, Ignacio Álvarez, Pau Pastor, José A. G. Agúndez

**Affiliations:** 1 Section of Neurology, Hospital Universitario del Sureste, E28500 Arganda del Rey, Spain; hortalon@yahoo.es; 2 ARADyAL Instituto de Salud Carlos III, University Institute of Molecular Pathology Biomarkers, University of Extremadura, E10071 Caceres, Spain; elenag@unex.es (E.G.-M.); jagundez@unex.es (J.A.G.A.); 3 Movement Disorders Unit, Department of Neurology, University Hospital Mútua de Terrassa, Fundació Docencia i Recerça Mútua de Terrassa, E08221 Terrassa, Spain; ignacioalvafer@gmail.com (I.Á.); pastorpau@gmail.com (P.P.)

**Keywords:** essential tremor, genetics, family history, linkage studies, genetic polymorphisms

## Abstract

There are many reports suggesting an important role of genetic factors in the etiopathogenesis of essential tremor (ET), encouraging continuing the research for possible genetic markers. Linkage studies in families with ET have identified 4 genes/loci for familial ET, although the responsible gene(s) have not been identified. Genome-wide association studies (GWAS) described several variants in *LINGO1*, *SLC1A2*, *STK32B*, *PPARGC1A*, and *CTNNA3*, related with ET, but none of them have been confirmed in replication studies. In addition, the case-control association studies performed for candidate variants have not convincingly linked any gene with the risk for ET. Exome studies described the association of several genes with familial ET (*FUS*, *HTRA2*, *TENM4*, *SORT1*, *SCN11A*, *NOTCH2NLC*, *NOS3*, *KCNS2*, *HAPLN4*, *USP46*, *CACNA1G*, *SLIT3*, *CCDC183*, *MMP10*, and *GPR151*), but they were found only in singular families and, again, not found in other families or other populations, suggesting that some can be private polymorphisms. The search for responsible genes for ET is still ongoing.

## 1. Introduction

Essential tremor (ET) is considered as one of the more prevalent movement disorders. Its main clinical feature is postural or kinetic tremor (or both combined), affecting exclusively or predominantly upper limbs, with a 4–12 Hz frequency [1,2]. A variable percentage of patients present tremor in other body regions (voice, head, tongue, trunk, and/or lower limbs) as well [1,2]. In addition to tremor, patients with probable or definite ET have shown impairment in several motor tasks, consistent with subtle bradykinesia [3,4,5,6,7,8], and many patients with ET also show associated co-morbidities or non-motor symptoms. These include depression [9,10,11,12,13,14,15,16], anxiety [9,10,11,12,13,14,15,16], cognitive impairment [9,10,11,12,13,14,15,16], fatigue [9,12,13,14,15,16,17,18], personality changes [10,11,13,15], olfactory dysfunction [9,10,11,13,14,15], hearing impairment [10,11,13,14,15], sleep disturbances [9,10,15,17,19,20,21], and upper airway dysfunction [22]. 

The etiopathogenesis of ET is not clearly established. Despite many reports in the literature suggesting an important role of genetic factors [23,24,25], these do not explain all cases, and a possible role of environmental factors has been suggested, especially to explain sporadic forms of ET [26,27,28]. The role of genetic factors in the etiology of ET is supported by the high frequency of positive family history of tremor in patients with ET, the description of genetic anticipation, that is, the onset of tremor at an earlier age in the next generation, and the higher concordance rates of ET for monozygotic than for dizygotic twins found in twin studies [23]. The most usual inheritance pattern of ET is an autosomal dominant mode (likely one or more autosomal dominant genes with low penetrance), though complex inheritance, autosomal recessive, X-linked patterns, and non-Mendelian patterns of inheritance have also been described [23].

Although a considerable effort has been made in recent years trying to identify genomic markers for ET, to date, the responsible genes have not been clearly established. In this review, we discuss studies addressing this issue, including linkage studies in families with ET, genome-wide association studies (GWAS) and exome sequencing studies in families and case-series of patients with ET, and hypothesis-driven case-control studies on candidate genes for this disease, updating previously reported information [23].

For this purpose, we performed a search using PubMed, Web of Science (main collection), and EMBASE databases from 1966 up to 6 April 2021, crossing the term “*essential tremor*” with “*genetics*” (784, 1004, and 411 items respectively, for PubMed, Web of Science, and EMBASE), “*genes*” (171, 681, and 298 items respectively, for PubMed, Web of Science, and EMBASE), “*risk factors*” (4, 421, and 320 items respectively, for PubMed, Web of Science, and EMBASE), “*linkage studies*” (58, 95, and 78 items respectively, for PubMed, Web of Science, and EMBASE), “*genome-wide association studies*” (60, 70, and 41 items respectively, for PubMed, Web of Science, and EMBASE), “*exome sequencing studies*” (19, 40, and 35 items respectively, for PubMed, Web of Science, and EMBASE), “*transcriptomic studies*” (3, 5, and 5 items), and “*case-control association studies*” (197, 70, and 55 items respectively, for PubMed, Web of Science, and EMBASE). The whole search retrieved a total of 1085 papers, that were examined manually. Then, those strictly related to the issue of genomic markers and ET were selected, excluding those in abstract form, and without language restrictions. 

## 2. Linkage Studies

Linkage analysis is a genetic tool that searches for physical segments of the genome that co-segregate with certain phenotypes or traits through families. These type of studies identified 4 susceptibility loci for familial ET, which have been located at chromosomes 3q13 [29], 2p25-p22 [30], 6p23 [31], and 5q35 [32]. The results of linkage studies in families with ET are summarized in Table 1.

The first locus linked to ET, named *FET1* (familial ET1) or *ETM1*, was reported in Icelandic families through a genome-wide scan study [29]. Linkage of ET to the *ETM1* gene was found in only 4 of 30 ET families of Slavonic and Tajik origin [34], but was not confirmed in studies of ET families of other geographical origins [33,35,36]. However, further studies described an association between the rs6280 SNP in the *dopamine receptor D3* (*DRD3*) gene (MIM/gene ID 126451/18149), which is responsible of the Gly9Ser amino acid change, and the risk for ET [43]. This gene is located in the *ETM1* locus and is currently designated as *ETM1* in the Gene Database. This variant was found in 23 of 30 French families with ET, and the presence of the *DRD3*Gly/Gly genotype was associated with an earlier ET onset [43]. Further association between the *DRD3*Gly allele with the risk for ET was found in two case-control association studies in North American [44] and Spanish populations [45]. However, the results of replication studies in other populations [46,47,48,49,50] and the lack of segregation in other families with ET [51,52] did not confirm such an association. The analysis of pooled results of case-control association studies showed a non-significant trend towards a slight overrepresentation of the *DRD3*Gly allele in ET patients compared with controls [23,53,54].

Regarding the *ETM2* gene, the initial linkage found in a large American-Czech family with “pure” autosomal dominant ET [30] was confirmed by the same research group in other independent American families [37] and a case-control association study [38]. Additionally, a decrease in short tandem repeats in the *ETM2* gene, designated as *ETM1234 microsatellite*, was found to be associated with the risk for ET in the Korean population [41]. In contrast, studies in other populations failed to find any linkage between ET and the *ETM2* locus in different populations [33,34,35,36,42]. Among the possible candidate genes included in locus *ETM2*, the rs11680700 variant within the *HS1BP3* gene (*HCLS1 binding protein 3*, MIM 609359, gene ID 64342) was described in two families with ET [55], and in 12 of 73 (16.4%) patients with familial ET unrelated among them, while this variant was absent in 304 healthy controls [55,56]. This variant was infrequent in 2 families with 27 members affected by ET (it was only present in 3 subjects of the same family) [57] and was not associated with the risk for ET in a case-control association study [58]. Linkage of ET to the *ETM3* locus found in a study of large American families [31] was not confirmed in Italian families [35,36]. Finally, linkage of ET was reported in 1 of 5 families at a locus on chromosome region 5q35, but to our knowledge, there are no replication studies [32].

## 3. Genome-Wide Association Studies (GWAS)

GWAS consist in analyzing many common spaced genetic variants in cases and controls trying to look for genetic markers that are associated with a disease. Identification of genetic markers can be useful to understand the contribution of genes to the risk of disease and to develop strategies for its prevention and therapy.

The first two GWAS reported, respectively, an association of the risk for ET with two SNPs in the *Leucine rich repeat and Ig domain containing Nogo receptor interacting protein-1* gene (*LINGO1*) [59,60], and with an intronic variant in the *solute carrier family 1-glial affinity glutamate transporter-, member 2* (*SLC1A2*) gene with ET [60]. However, further replication studies on these variants showed controversial results, that will be discussed in the next section.

### 3.1. Studies on LINGO Gene Family

The first GWAS described a strong statistical association between the intronic rs9652490 and rs11856808 SNPs in the *LINGO1* gene (chromosome 15q24.3, MIM 609791, gene ID 84894) and the risk for ET in the Icelandic population, but, after adjusting for the rs9652490 genetic effect, the association with rs11856808 disappeared, and the only confirmed in diverse studies was that of rs9652490 with ET [59]. However, LINGO1 should be an interesting candidate gene to modify risk of ET by many reasons, which are outside of the scope of the present review [23,61], and even could be potentially related to the therapy of this disease [61].

The effect of the rs9652490 variant in the susceptibility for ET was replicated in 5 studies [62,63,64,65,66], but not in another 5 [67,68,69,70,71]. In addition, the association of ET with rs11856808 was replicated by one group [60], but not by another 5 groups [67,68,69,70,71].

A meta-analysis found no association whatsoever for the rs9652490G allele, and identified a weak association with the risk of developing ET and the presence of the rs11856808T allele [72]. Nevertheless, both rs9652490G and rs11856808T alleles had a weak association with ET limited to patients with a positive family history of ET [72], whereas two other meta-analyses described association [54] and lack of association [73] between rs9652490 SNP and ET risk. Interestingly, some authors reported a significant association of the *LINGO1* rs9652490AA genotype and the rs9652490A allele with ET risk, under a recessive model, in a North American series [63,65], while the association described by other authors was with the minor allele rs9652490G [59,60,62,66]. This flip-flop phenomenon [74] could have affected the results of the meta-analyses. Moreover, a further two-stage GWAS involving 2807 ET patients and 6441 controls of European descent did not find an association with rs9652490 [75].

Other variants in the *LINGO1* gene have been reported to be associated with the risk for ET or to an earlier onset of ET in single studies. An association between the rs8030859T allele and the risk for ET was reported in the German population [60], and a weak association between the rs7177008, 13313467, and rs8028808 and early-onset ET was reported in North Americans [66]. A case-control association study in Chinese Han did not find any association of rs2271398, rs2271397, rs3743481, and a novel G → C transition ss491228439 SNPs variants and the risk for ET, although rs2271397 and ss491228439 variants could contribute to the risk for ET among females [76].

A study in North Americans described an association of 5 tagging SNPs within, or close to, the *LINGO1* and *LINGO2* (MIM 609793, Gene ID 158038) genes (rs4886887, rs3144, rs8028808, rs12905478, and rs1412229) with the risk of developing ET [63,65], and another study involving Asian populations found an association of the *LINGO2* rs7033345CC genotype and the *LINGO2* rs10812774C allele with the risk for ET under a recessive model [77]. Finally, another study in Chinese Han found a lack of association between the rs61746299 and rs1521179 SNPs in the *LINGO4* gene (MIM 609794, Gene ID 339398) and ET risk [78].

### 3.2. Studies on SLC1A2 Gene

The *SLC1A2* gene (*solute carrier family 1-glial affinity glutamate transporter-member 2*, chromosome 11p13-p12, MIM 600300, Gene ID 6506) encodes a member of a family of solute transporter proteins, one of them being the main transporter of the excitatory neurotransmitter glutamate. This neurotransmitter plays an important role in the pathogenesis of ET [79], although a detailed description of this issue is outside of the scope of this review.

The second GWAS described a strong association (odds ratio (95% CI) = 1.59 (1.36–1.84)) between the SNP rs3794087 in the *SLC1A2* gene and the risk for definite ET in a GWAS of 990 patients with ET (658 with definite ET) and 1490 healthy controls [60]. This variant could be an interesting genetic marker for ET, since one study has replicated this association in the Chinese [80] and Taiwanese populations [81]. However, this association was not confirmed in additional replication studies [82,83,84], two meta-analyses [54,85], and was not found in several families with ET [52], nor in two-stage GWAS involving 2807 ET patients and 6441 controls of European descent [75].

### 3.3. Results of Other GWAS Studies

A two-stage GWAS involving 2807 ET patients and 6441 controls of European descent reported association of ET with two markers: the intronic SNP rs10937625 in the *serine*/*threonine kinase 32B* gene (*STK32B*, chromosome 4p16.2, Gene ID 55351; the protein encoded by this gene participates in the transfer of phosphate molecules to the oxygen atoms of serine and threonine), and rs17590046 in the *PPARG coactivator 1 alpha* gene (*PPARGC1A*, chromosome 4p15.2; MIM 604517, gen ID 10891; PPARGC1 protein acts as a transcriptional coactivator that regulates the genes involved in energy metabolism) [75]. In addition, in a combined analysis, this study found a significant association of the markers rs12764057, rs10822974, and rs7903491 in the *catenin alpha 3* gene (*CTNNA3*, chromosome 10q21.3, MIM 607667, Gene ID 29119; CTNNA3 plays a role in cell–cell adhesion in muscle cells) [75]. Interestingly, this study also described increased expression of STK32B in the cerebellar cortex of ET patients and association between the minor allele of rs10937625 and reduced expression of STK32B in the cerebellar cortex [75].

A replication study in Asian patients involving 469 ET patients and 470 controls confirmed the association of ET with *PPARGC1A* rs17590046, but not with the *STK32B* rs10937625 variant [86]. A Canadian study did not find any rare exonic variant in *STK32B*, *PPARGC1A*, and in *CTNNA3* genes in a whole-exome and whole-genome sequencing study involving 14 autosomal-dominant multiplex ET families and in a targeted massive parallel sequencing study of these 3 genes in 269 ET patients and 287 controls [87].

An estimation of narrow-sense heritability by using the genomic-relationship matrix restricted maximum likelihood (GREML-LDMS) to measure the phenotypic variance explained by genetics in a study involving 1751 ET cases and 5311 controls showed that ET is a highly heritable condition with an important role of common variability, with chromosomes 6 and 21 being those that contained potential causative risk variants influencing genetic susceptibility to ET [88].

## 4. Exome and Whole-Genome Sequencing Studies

Exome sequencing of whole-exome sequencing is an efficient strategy to selectively sequence the exome (that is, all the protein-coding regions of the genome). Whole-genome sequencing is the process of determining the complete DNA sequence (both nuclear and mitochondrial DNA) of all of the genome with a single run. During the last decade, there have been an important number of contributions reported looking for genetic markers for ET through exome sequencing and increasing studies even using whole-genome sequencing.

### 4.1. Fused in Sarcoma/Translated in Liposarcoma (FUS/TLS or FUS) Gene

The first whole-exome sequencing published in familial ET found the p.Q290X mutation (rs387907274) in the *fused in sarcoma*/*translated in liposarcoma* gene (*FUS*/*TLS*, *FUS* or *FUS RNA protein*, currently designated as *ETM4*, chromosome 16p11.2, MIM 137070, gene ID 2521; this gene encodes a protein which is component of a heterogeneous nuclear riboprotein, which is involved in pre-mRNA splicing and the export of fully processed mRNA to the cytoplasm). Mutations of this gene were previously described in families with amyotrophic lateral sclerosis and frontotemporal dementia. The *FUS* p.Q290X mutation segregated with ET in a large Canadian family, and two rare missense variants (p.R216C-rs267606832 and p.P431L-rs186547381) were found in a further screening of 270 ET cases [89]. Interestingly, some authors generated a transgenic model in Drosophila expressing hFUS-WT and hFUS-Q290X and found that expression of hFUS-Q290X caused a motor dysfunction linked to the impairment in the GABAergic neurotransmission which was partially rescued with gabapentin [90].

However, many further studies did not find pathogenic mutations in the *FUS* gene [91,92,93,94,95,96] or only described extremely rare novel risk variants causing amino acid substitutions, such as Met392Ile (rs751937417) [97] or p.R377W (rs766187715) [98]. An association between the synonymous coding SNP *FUS* rs1052352 and the risk for ET has been described in the Chinese population [99].

The presence of a gene variant causing the amino acid exchange R471C substitution in the gene *EWSR1* (*EWSR1 binding protein*, chromosome 22q12.2, MIM 133450, Gene ID 2130) has been reported, related with *FUS* (as genes encoding RNA-binding proteins), in a single subject with familial ET from two subsets of ET patients (*n* = 661) and controls (*n* = 886) [100].

### 4.2. Mitochondrial Serine Peptidase 2 (HTRA2) Gene

A study in a six-generation consanguineous Turkish kindred identified the p.G399S mutation (rs72470545) in the *HtrA serine peptidase 2* (*HTRA2*, chromosome 2p13.1, MIM 606441, Gene ID 27429; this gene encodes a serine protease localized in the endoplasmic reticulum and in the mitochondria that is released and has a role in apoptosis, and it is suggested to be involved in familial Parkinson’s disease (PD), designated as the *PARK13* gene as well), as it is proposed to be responsible for both ET and PD [101]. ET was present both in heterozygous or homozygous for this allele, while only homozygotes developed PD, and homozygosity was related with earlier disease onset and higher severity of tremor [101]. However, further studies in different populations showed that *HTRA2* mutations were very infrequent or absent [99,102,103,104,105,106].

### 4.3. Teneurin Transmembrane Protein 4 (TENM4) Gene

A whole-exome sequencing followed by targeted resequencing found missense mutations in the *teneurin transmembrane protein 4* gene (*TENM4*, *ETM5*, chromosome 11q14.1, MIM 610084, Gene ID 26011, TENM4 protein plays a role in establishing proper neuronal connectivity during development, and is a regulator of axon guidance and central myelination), and showed that *TENM4* variants segregated in an autosomal-dominant fashion in three Spanish families with ET [107]. However, studies in 3 cohorts of ET patients and controls detected several missense variants in both groups, but the allele frequencies did not differ significantly among ET patients and control groups [99,108,109]. Interestingly, an ET phenotype has been reported in *Tenm4* knockout mice [110].

### 4.4. Sortilin 1 (SORT1) Gene

A whole-exome sequencing and subsequent approaches including functional analysis, in a Spanish family with an autosomal-dominant form of early-onset ET, described a disease-segregating mutation p.Gly171Ala (rs750957839), that was absent in the normal population, in the *sortiline 1* gene (*SORT1*, chromosome 1p21.3-p13.1, MIM 602458, Gene ID 6272; this gene encodes a member of the VPS10-related sortilin family of proteins which are proteolytically processed by furin to generate the mature receptor, that plays a role in the protein trafficking to either the cell surface or subcellular compartments such as lysosomes and endosomes) [111]. The p.Gly171Ala variant impaired the expression of sortilin and decreased mRNA levels of its binding partner p75 neurotrophin receptor implicated in neurotransmission, neuronal apoptosis, and brain injury [111].

### 4.5. Sodium Voltage-Gated Channel Alpha Subunit (SCN11A) Gene

A whole-exome sequencing in a four-generation Chinese family with early-onset familial episodic pain and adult-onset familial ET showed the missense mutation p.Arg225Cys (rs138607170) in the *sodium voltage-gated channel alpha subunit* gene (*SCN11A*, chromosome 3p22.2, MIM 604385, Gene ID 11280; SCN11A is a transmembrane glycoprotein complex composed of a large alpha subunit with 24 transmembrane domains and one or more regulatory beta subunits that are responsible for the generation and propagation of action potentials in neurons and muscle. SNC11a proteins are highly expressed in the nociceptive neurons of dorsal root ganglia and trigeminal ganglia and participate in peripheral inflammatory pain hypersensitivity) [112]. The authors suggested that, according to these findings, ET should be considered as a channellopathy.

### 4.6. Notch 2 N-Terminal-Like (NOTCH2NLC) Gene

A study using a research strategy that combined linkage analysis, whole-exome sequencing, long-read whole-genome sequencing, repeat-primed polymerase chain reaction, and GC-rich polymerase chain reaction, in 197 Chinese pedigrees with ET, identified in 11 of them (co-segregating with the disease) an abnormal CGG repeat expansion in the 5′ region of the *Notch 2 N-terminal-like* gene (*NOTCH2NLC* or *ETM6*, chromosome 1q21.2, MIM 618025, Gene ID 100996717, mutations in this gene are associated with neuronal intranuclear inclusion disease, or NIID) [113]. Subjects carrying this mutation had higher severity of tremor, and these 11 families showed genetic anticipation [113]. This gene has been designated as *ETM6*.

Other authors described abnormal CGG repeat expansions (>60) in the *NOTCH2NLC* in Asiatic patients with ET [114,115,116]. A whole-exome sequencing in 30 members from 15 Chinese families with ET (10 of them diagnosed with ET) found abnormal CGG repeat expansions in 16 subjects, 4 of them developed cognitive impairment, and 3 were finally diagnosed with NIID [114]. Another study identified pathogenic *NOTCH2NLC* CGG expansions in 4 of 285 Singaporean individuals with sporadic ET (one of them developed motor and cognitive impairment 8–10 years later) and in none of 125 ET patients with a family history of ET, in 52 probands from ET pedigrees, and in 200 controls (although 4 patients with a family history of ET showed 47 to 53 “intermediate” repeats) [115]. Finally, there have been abnormal *NOTCH2NLC* CGG expansions found in 3 of 28 probands of families with ET [116].

In contrast, abnormal *NOTCH2NLC* CGG expansions are very rare in European ET patients. One group did not find any abnormal expansion in a series of 111 European patients with ET (74 with “pure” ET and 37 with ET-plus) [117], and only one mutation in another cohort of 203 ET patients [118], and another study did not find any abnormal expansion in 204 ET patients and 408 controls of European ancestry [119].

### 4.7. Results from Other Exome and Whole-Genome Sequencing Studies in ET Patients

A study using SNP arrays followed by whole-exome sequencing in a family with highly penetrant autosomal-dominant tremor (17 members, 5 of them affected with ET) did not identify any copy number variation or mutation related to the ET phenotype [120].

A whole-exome sequencing study involving 37 early-onset ET families with an autosomal-dominant inheritance pattern identified two heterozygous variants, p.Gly16Ser (rs368332097) and p.Pro55Leu (rs374957936), in the *nitric oxide synthase 3* or *endothelial NOS* gene (*NOS3*, chromosome 7q36.1, MIM 163729, Gene ID 44847) in 2 families co-segregating with the disease, and variants in other genes including the *potassium voltage-gated channel modifier subfamily S member 2* (*KCNS2*, *Chromosome 8q22.2*, MIM 602906, Gene ID 3788), *hyaluronan and proteoglycan link protein 4* (*HAPLN4*, chromosome 19p13.11, Gene ID 4040379), and *ubiquitin-specific peptidase* (*USP46*, chromosome 4q12, MIM 612849, Gene ID 64854), each of them in 3 other independent families [121]. All of these genes influence the GABAergic system function and have a high expression in the cerebellum [121].

A whole-genome sequencing study involving 40 individuals from 8 ET families identified the deleterious and damaging variant p.Arg456Gln (rs116920450) in the *calcium voltage-gated channel subunit alpha1 G* gene (*CACNA1G*, chromosome 17q21.33, MIM 604065, gene ID 8913; the T-type low-voltage activated calcium channel encoded by this gene generates transient currents, owing to fast inactivation, and tiny currents, owing to small conductance, and is thought to be involved in pacemaker activity, low-threshold calcium spikes, neuronal oscillations, resonance, and rebound burst firing) in one family, and a variant in the *slit guidance ligand 3* gene (*SLIT3*, chromosome 5q34-q35.1, MIM 603745, Gene ID 6586; the protein encoded by this gene acts as an axon guidance molecule) in another [122].

Finally, a study in 40 individuals from 8 families with autosomal-dominant ET by using whole-exome sequencing followed by a case-control association study comprising a total of 1310 ET patients and 1366 controls from two cohorts, looking for the association of rare variants with ET risk, found co-segregation with the disease in at least one family with the variants rs749875462, located in the *coiled-coil domain containing 183* (*CCDC183*, chromosome 9q34.3, MIM 615955, Gene ID 849609), rs535864157, located in the *matrix metalopeptidase 10* (*MMP10*, chromosome 11q22.2, MIM 185260, Gene ID 4319), and rs114285050, located in the *G protein-coupled receptor 151* genes (*GPR151*, chromosome 5q.32, MIM 618487, Gene ID 134391) [123]. MMP10 protein belongs to the peptidase M10 family of matrix metalloproteinases, which are involved in the breakdown of extracellular matrix in normal physiological processes, such as embryonic development, reproduction, and tissue remodeling. GPR151 protein belongs to a class of rhodopsin-like family of G-protein-coupled receptors, which also includes somatostatin, opioid, galanin, and kisspeptin receptors. However, the frequency of these variants was very low both in ET patients and in controls in the replicatory case-control association study [123].

## 5. Transcriptomic Studies

Transcriptomics technologies are used to study an organism’s transcriptome, that is, the sum of all of its RNA transcripts. For the first transcriptomic analysis by direct sequencing of RNA from frozen cerebellar cortex tissue in 33 ET patients compared to 21 normal controls, differential expression analysis between ET patients and controls identified 231 differentially expressed gene transcripts, that contributed to the regulation of axon guidance, microtubule motor activity, endoplasmic reticulum to Golgi transport, and calcium signaling/synaptic transmission [124].

A case-control RNA-sequencing study analyzing cerebellar cortex and dentate nuclei from 16 ET patients and 16 controls, using a multi-omics approach (phenome-wide association study, or pheWAS, genome-wide gene association study, or GWGAS, and transcriptome-wide association study, or TWAS) reported differences in the expression of several genes in ET patients compared with controls (*PRKG1*-kinase function, *SAC3D1*-mitotic function, *SHF*-apoptotic function, *TRAPPC11*-protein trafficking function, *NELL2*-neuronal survival function, and *CACNA1A*-calcium channel function in the cerebellar cortex, and *PLCG2*-phospholipase and *ALDH3A2*-dehydrogenase in the dentate nucleus) [125].

A study with human cerebellar DAOY cells with overexpression of STK32B RNA using an RNA-Seq approach to identify differentially expressed genes (DEGs), by comparing the transcriptome profile of these cells to one of the control DAOY cells, identified dysregulation in several potentially relevant ET genes, including *FUS*, *CACNA1C*, and *CACNA1A*, and differentially expressed genes including olfactory transduction, axon guidance, and calcium ion transmembrane transport genes [126].

## 6. Studies on Candidate Genes

Postural and intention tremors are frequently observed in many neurological diseases, such as PD, dystonia, and spinocerebellar ataxias (SCAs), among others. For this reason, in an attempt to search for common etiological factors, many researchers have analyzed the possible role of genes related with some of these diseases in the risk for ET. In fact, the relationship between ET and PD is supported by many epidemiological, genetic, clinical, neuropathological, and neuroimaging data [127,128]. For this reason, many studies looking for possible genomic markers for ET analyzed both genes showing an association with PD in hypothesis-driven case-control association studies or genes related with monogenic familial PD. Table 2 summarizes the results of studies on candidate genes for ET that had been previously related to PD. Although a number of studies showed weak associations between several variants in certain genes with the risk for ET, replication studies did not confirm these associations. Replication studies on the possible contribution of several allelic variants in the genes *CYP2C19*, *CYP2C9/CYP2C8*, *RIT2*, and *IL1B*, which have shown an association with the risk for ET in single studies, are lacking.

Table 3 summarizes the results of studies on other candidate genes, not related to PD, in the risk for ET. In summary, a lack of association of genes have been described related with idiopathic torsion dystonia [34,165,166], spinocerebellar ataxias [167,168,169,170], and fragile X-associated tremor/ataxia syndrome (FXTAS) [171,172,173,174,175,176,177], with genes related with potassium and sodium channels [167,168], GABAergic pathways, calcium and glutamate signaling pathways, and with mitochondrial genes.

Interestingly, despite that the rs1800435 variant in the *amino-levulinic acid dehydratase* (*ALAD*) gene has not shown a direct association with ET risk in two studies [178,179], this variant showed association with this risk in interaction with serum lead levels [178] and with the *heme-oxygenase 2* (*HMOX2*) rs1051308G variant [179].

Firstly, a *knockout gamma-aminobutyric acid type A receptor subunit alpha1 GABR Alpha1* (*GABRA1*) mouse showed postural and kinetic tremor and motor un-coordination [188], and, secondly, a *knockout* mouse for the *GABA transporter* (*GAT*)-*1* gene showed a motor disorder, including a 25 to 32 Hz frequency tremor [189]. However, three case-control association studies failed to find any association between *GABR* or *GAT* genes and the risk for ET [182,183,184].

Since ET is associated with restless leg syndrome [190], two case-control association studies have addressed the possible role of genes previously related to restless leg syndrome in the risk for ET. The initial description of an association of the rs6494696 SNP and a haplotype (rs4489954, rs3784709, rs2241420, rs1026732, and rs6494696) with the risk for ET [185] was not replicated by other study [186].

The tremulous dominant mutant Kyoto rat is a rat model that shows a spontaneous tremor resembling human ET. Recent studies have identified a missense mutation (c.1061 C>T, p. A354V) in the *hyperpolarization-activated cyclic nucleotide-gated potassium 1 channel* (*Hcn1*) gene [191], and a missense substitution (c. 866T>A, p. I289N) in the *potassium calcium-activated channel subfamily N member 2* (*Kcnn2*) gene in this model [192]. To our knowledge, associations of variants in the equivalent human genes (*HCN1*, chromosome 5p12, MIM 602780, Gene ID 348980, and *KCNN2*, chromosome 5q22.3, MIM 605879, Gene ID 3781) with the risk for ET in humans have not been analyzed to date. A study reported the lack of association between CAG repeat expansions in the *KCCN3* gene and the risk for ET [167].

Finally, a deletion in the class II ADP ribosylation factor genes (ARF4 and ARF 5) in mice (ARF4+/−/ARF5−/−) causes Nav1.6 loss in cerebellar Purkinje cell axon initial segments and ET-like behaviors [193]. To our knowledge, variants in the equivalent human genes (ARF4, chromosome 3p14.3, MIM 601177, Gene ID 374; ARF5, chromosome 7q32.1, MIM 103188, Gene ID 381) have not been studied to date.

## 7. Conclusions and Future Directions

Despite that genetic factors have an important role in ET, the search for the responsible gene(s) is still ongoing. The identification of 4 genes/loci in several families through linkage studies (the 3 first reported designated as *ETM1*, *ETM2*, and *ETM3* genes) has not been confirmed in other family studies, and, moreover, they should only explain a small percentage of familial ET, and the responsible genes remain to be identified. As it was previously mentioned, genetic factors do not explain all cases of ET, and recent studies have suggested a possible role of several environmental factors such as *β*-carboline alkaloids and ethanol, agricultural work, pesticide, lead, and harmanes, with antioxidant intake and smoking being possible protective agents [27,28,194,195,196].

Regarding treatment of ET, the drugs that have shown higher efficacy are the beta-blocker propranolol and the antiepileptic drug primidone, but other drugs such as 1-octanol and octanoid acid, and drugs acting on the glutamatergic system, the extra-synaptic GABAA receptors, or LINGO-1 could be interesting therapeutic options, and injections of botulinum toxin A have shown to be useful in the treatment of refractory ET [197].

The results of GWAS studies reported to date have not been conclusive. Despite the results of the first GWAS that pointed to 2 *LINGO1* variants, further case-control association studies showed a weak association of these variants with the risk for familial ET, and a further GWAS failed to replicate the findings [75]. The results of the second GWAS suggested the association of the rs3794087 variant in the *SLC1A2* gene with ET but, again, these were not replicated [54,72,73,75]. The role of several variants in the *STK32*, *PPARG1A*, and *CTNNA3* genes suggested by the third GWAS [75] has not been confirmed by other groups [87], except for *PPARG1A* in the Chinese population [86].

Exome and whole-genome sequencing studies have found several candidate variants possibly responsible for ET in a small number of families, in split genes such as *FUS* (designated as *ETM4*), *HTRA2*, *TENM4* (designated as *ETM5*), *SORT1*, *SCN11A*, *NOTCH2NLC* (designated as *ETM6*), *NOS3*, *KCNS2*, *HAPLN4*, *USP46*, *CACNA1G*, *SLIT3*, *CCDC183*, *MMP10*, and *GPR151.* However, replication studies on *FUS*, *HTRA2*, *TENM4*, and *NOTCH2NLC* genes have found that these mutations are infrequent in other families and populations, while results on mutations of other genes remain to be replicated.

Finally, candidate gene studies have not identified an association with ET risk for genes previously related with other degenerative diseases such as PD, idiopathic torsion dystonia, hereditary ataxias, or others, except for the findings on several variants of *CYP2C19* [137], *CYP2C9*/*CYP2C8* [145], *RIT2* [158], and *IL1B* [147], and the increased risk for carriers of the *ALAD* rs1800435 variant in interaction with serum lead levels [178] or with a variant in the *HMOX2* gene [179]. However, the results of these studies have not been replicated so far.

Several factors should be taken into account as limitations in the investigation of genomic markers for ET, such as the lack of disease-specific non-genetic markers for ET (the diagnosis is done on clinical grounds), its frequent overlap with other disorders such as dystonia and PD, and the possible inclusion of phenocopies in genetic studies [23]. Table 4 summarizes the minimal conditions that should be fulfilled by studies trying to address genomic markers for ET.

## Figures and Tables

**Table 1:** Results of linkage studies in patients with familial essential tremor (ET).

Country	Locus	Chromosome	MIM/ Gene ID	Study Subjects	Main Findings/Comments	[Ref]
Iceland	*ETM1* (*FET1*)	3q13	190300/2111	Genome-wide scan study involving 16 Icelandic families (75 affected individuals) with ET in an autosomal dominant pattern	Gene mapped at chromosome 3q13 with a genome-wide significance assuming an autosomal-dominant model (parametrically LOD score = 3.71 and non-parametrically LOD score = 4.70). The highest single-family LOD score was 1.29	[29]
United States of America	*ETM1* (*FET1*)	3q13	190300/2111	Linkage analysis with microsatellite markers for *ETM1*, *ETM2*, and chromosome 4p in 38 members of a six-generation family with ET	Lack of association with the analyzed loci, including *ETM1* and with chromosome 4p	[33]
Russia	*ETM1* (*FET1*)	3q13	190300/2111	Linkage analysis for *ETM1* and *ETM2* loci, and for locus *DYT1* on chromosome 9q32-34 in a group of Slavonic (11 patients) and Tajik (19 patients) families with ET.	Linkage to locus *ETM1* in 4 families (maximum pairwise LOD score 2.46, maximum combined multipoint LOD score was 3.35 for marker D3S3720) and a common “mutant” haplotype for markers D3S3620, D3S3576, and D3S3720 in a 2 Cm chromosomal region	[34]
Italy	*ETM1* (*FET1*)	3q13	190300/2111	Linkage analysis of 3 loci previously associated with ET (*ETM1*, *ETM2*, and a locus on 6p23 (*ETM3*)) in fifth-generation Italian kindred with autosomal-dominant ET (22 clinically evaluated family members, 9 were affected by ET).	Lack of association with *ETM1*	[35]
Italy	*ETM1* (*FET1*)	3q13	190300/2111	Linkage analysis for *ETM1*, *ETM2*, and *ETM3* in a large family with autosomal-dominant ET involving 6 generations	Lack of association with *ETM1*	[36]
United States of America	*ETM2*	2p25-p22	602134/2112	Linkage analysis in a large American-Czech family with “pure” autosomal-dominant ET (138 members, with 18 affected with ET; genetic anticipation over generations)	This gene was mapped close to D2S272 at chromosome 2p25-p22 (maximum LOD score = 5.92). Affected relatives showed a CAG repeat expansion not clearly located in the *ETM2* locus.	[30]
United States of America	*ETM2*	2p25-p22	602134/2112	Linkage analysis in the previous large American-Czech family with “pure” autosomal-dominant ET and in 3 additional, unrelated American families using fine mapping results in an “only-affected” model	Positive combined pairwise LOD scores (Z) at the *ETM2* locus with a Z(max) = 5.94 at a recombination fraction (theta) = 0.00 for locus D2S220. Haplotype reconstruction places the ETM2 gene in a 9.10 cM interval (D2S224-D2S405) Multipoint linkage analysis suggested that the *ETM2* gene was a the 2.18 cM interval (D2S2150 and D2S220; Z(max) = 8.12).	[37]
United States of America	*ETM2*	2p25-p22	602134/2112	Linkage disequilibrium study involving 45 patients with familial ET and 70 normal controls (*n* = 70). Identification of 3 unreported dinucleotide polymorphic loci designated as etm1240, etm1231, and etm1234 in the *ETM2* gene and haplotype analysis	Significant differences in the allele frequencies between ET and controls of etm1231 (*p* = 0.0419) and etm1234 (*p* < 0.0001) loci. Significantly higher frequency of the A haplotype formed by the loci etm1231 and etm1234 in ET patients than in controls (29% vs. 9%)	[38]
United States of America	*ETM2*	2p25-p22	602134/2112	Linkage disequilibrium study involving 52 Singaporean patients with familial ET and 49 Singaporean normal controls (*n* = 70). Analysis of 6 polymorphic loci (etm1240, etm1231, etm1234, APOB, etm1241, and etm1242) in a 274 kb interval within an ET gene candidate region (ETM2), including haplotype analysis.	Significant differences in the allele frequencies between cases and controls for the loci etm1234 (*p* = 0.0001) and APOB (*p* = 0.0320). Significantly higher frequency of a haplotype formed by the loci etm1231, etm1234, and APOB in ET patients than in controls (31% vs. 1.8%, *p* = 0.0005).	[39]
United States of America	*ETM2*	2p25-p22	602134/2112	Assembling of a physical map of the region between D2S224 and D2S2221 in the *ETM2* locus by using high-throughput non-isotopic screening of bacterial artificial chromosomes (BACs), and construction of a complementary integrated physical map of the human *ETM2* identifying GenBank contigs that contained seven BAC DNA sequences and common STSs.	Identification of 33 transcripts including five known genes (*MATN3*, *LAPTM4A*, *SDC1*, *PUM2*, and *APOB*) in this minimal critical region.	[40]
United States of America	*ETM2*	2p25-p22	602134/2112	Linkage analysis with microsatellite markers for *ETM1*, *ETM2*, and chromosome 4p in 38 members of a six-generation family with ET	Lack of association with *ETM2* and with chromosome 4p	[33]
Russia	*ETM2*	2p25-p22	602134/2112	Linkage analysis for *ETM1* and *ETM2* loci, and for locus *DYT1* on chromosome 9q32-34 in a group of Slavonic (11 patients) and Tajik (19 patients) families with ET.	Lack of association with *DYT1* and *ETM2* loci.	[34]
Korea	*ETM2*	2p25-p22	602134/2112	Genetic association with 3 polymorphic loci (STS-etm1240, STS-etm1231, and STS-etm1234) located in a region of the *ETM2*, in 30 ET patients and 30 controls.	Detection of 8 different sequence variants (5 at etm1234, 2 at etm1240, and 1 at etm1231) in 7 patients (only in patients with “classic ET”). Decrease in the number of short tandem repeats within etm1234 locus more frequently in ET patients than in controls.	[41]
Italy	*ETM2*	2p25-p22	602134/2112	Linkage analysis for *ETM1*, *ETM2*, and *ETM3* in a fifth-generation Italian kindred with autosomal-dominant ET (22 clinically evaluated family members, 9 were affected by ET).	Lack of association with *ETM2* locus	[35]
Italy	*ETM2*	2p25-p22	602134/2112	Linkage analysis for *ETM1*, *ETM2*, and *ETM3* in a large family with autosomal-dominant ET involving 6 generations	Lack of association with *ETM2* locus	[36]
Czech Republic	*ETM2*	2p25-p22	602134/2112	Genetic analysis of 3 polymorphic loci (etm1231, etm1234, and etm1240), located within the *ETM2* locus, in 61 Czech patients with familial ET and 68 healthy controls.	Lack of association with *ETM2* locus	[42]
United States of America	*ETM3*	6p23	611456/101027378	Genome-wide linkage screening and fine mapping in seven large North American families (325 individuals, 65 of them with definite ET).	Linkage to a locus on chromosome 6p23 in a family. A second family showed linkage to the same 6p23 region with a maximal NPL score 2.125 (*p* = 0.0075) and LOD score 1.265. Haplotype analysis led to the identification of a 600 kb interval shared by both families. Sequencing of 15 candidate genes located within this region did not find any sequence variants with pathogenic significance	[31]
Italy	*ETM3*	6p23	611456/101027378	Linkage analysis for *ETM1*, *ETM2*, and *ETM3* in a fifth-generation Italian kindred with autosomal-dominant ET (22 clinically evaluated family members, 9 were affected by ET).	Lack of association with *ETM3*	[35]
Italy	*ETM3*	6p23	611456/101027378	Linkage analysis for *ETM1*, *ETM2*, and *ETM3* in a large family with autosomal-dominant ET involving 6 generations	Lack of association with *ETM3*	[36]
United States of America	No specific name	5q35	-	Linkage analysis using an affected-only dominant model involving 48 ET patients who belonged to 5 large ET pedigrees. Identification of genome segments followed by exome sequencing in pedigrees showing evidence of linkage.	One family showed genome-wide significant linkage to ET in chromosomes 5 and 18, but shared segment analysis reduced the 5q35 region by 1 Mb, and excluded the 18p11 candidate region. No causative variants in the 5q35 region were identified after exome sequencing.	[32]

**Table 2:** Results of case-control association studies and case-only studies of possible candidate genes for ET.

Country	Gene	Chromosome	MIM/ Gene ID	Allelic Variant/ Mutations	Study Participants	Main Results	[Ref]
Spain	*Cytochrome P450 family 2 subfamily D member 6* (*CYP2D6*)	22q13.2	124030/1565	CYP2D6 other than *1 (*wild type*)	91 ET patients and 258 controls	Lack of association with ET	[129]
United States of America	*Synuclein alpha* (*SNCA*, *PARK1*)	4q22.1	163890/6622	257, 259, 261, and 263 bp alleles of non-amyloid component of plaques (NACP-Rep1, promoter region)	46 ET patients and 100 controls	Association between allele 263 bp and risk for ET, with an OR (95% CI) = 6.42 (2.04–21.4)	[130]
Italy	*Synuclein alpha* (*SNCA*, *PARK1*)	4q22.1	163890/6622	Several SNPs (intronic region between exons 1 and 2, NACP-Rep1)	106 ET patients and 90 controls	Lack of association with ET	[131]
United States of America	*Synuclein alpha* (*SNCA*, *PARK1*)	4q22.1	163890/6622	20 variants in the SNCA locus	661 ET patients and 1316 controls	Lack of association with ET	[132]
Italy	*Parkin RBR E3 ubiquitin protein ligase* (*PRKN*, *PARK2*)	6q26	602544/5071	Point mutations in the coding region of the gene	110 ET patients	Detection of 2 previously reported polymorphisms and 4 novel rare variants located within exonic regions, and 4 new polymorphisms and 1 rare variant within intronic regions, but all of them were not causative	[133]
Turkey	*Methyl-tetrahydrofolate reductase* (*MTFHR*)	1p36.22	607093/4524	rs1801133 rs1801131	158 ET patients and 246 controls	Individuals with T677T or T677T/A1298A genotypes have even greater susceptibility to essential tremor. Nevertheless, individuals with C677C/A1298A and C677T/ A1298A genotypes had a protective effect on essential tremor. Individuals with T677T or T677T/A1298A genotypes have even greater susceptibility to essential tremor. Nevertheless, individuals with C677C/A1298A and C677T/ A1298A genotypes had a protective effect on essential tremor. Increased risk for ET in carriers of the T677T or T677T/A1298A genotypes and decreased risk in those with C677C/A1298A and C677T/A1298A genotypes	[134,135]
China	*Methyl-tetrahydrofolate reductase* (*MTFHR*)	1p36.22	607093/4524	rs1801133 rs1801131	200 ET patients and 430 controls (Chinese Han)	Lack of association with ET	[135]
China	*Alpha2-macroglobulin* (*A2M*)	12p13.31	103950/2	A2M1000G (rs669)	73 ET patients and 100 controls	Lack of association with ET	[136]
Spain	*Cytochrome P450 family 2 subfamily C member 19* (*CYP2C19*)	10q23.33	124020/1557	CYP2C19 *1, *2, and *3	200 ET patients and 300 controls	Association of genotype CYP2C19*1/CYP2C19*2 and allelic variant CYP2C19*2 with ET risk. Lack of association with adverse effect by primidone	[137]
United States of America	*Leucine-rich repeat kinase 2* (*LRRK2*, *dardarin*, *PARK8*)	12q12	609007/120892	G2019S (rs34637584), I2012T (rs34015634), and I2020T mutations in the MAPKKK domain (exon 41)	272 ET patients	Lack of detection of mutations	[138]
Singapore	*Leucine-rich repeat kinase 2* (*LRRK2*, *dardarin*, *PARK8*)	12q12	609007/120892	Gly2385Arg (rs34778348)	172 ET patients and 247 controls	Lack of association with ET (frequency of minor allele 2.9% vs. 4.0%)	[139]
Italy	*Leucine-rich repeat kinase 2* (*LRRK2*, *dardarin*, *PARK8*)	12q12	609007/120892	G2019S (rs34637584), I2012T (rs34015634), and I2020T mutations in the MAPKKK domain (exon 41)	116 patients with familial ET	Lack of detection of mutations	[140]
United States of America	*Leucine-rich repeat kinase 2* (*LRRK2*, *dardarin*, *PARK8*)	12q12	609007/120892	4 LRRK2 mutations (G2019S (rs34637584), I2020T, R1441C (rs33939927), and Y1699C), 2 rare LRRK2 variants (L1114L and I1122V), and 19 LRRK2 SNPs	275 ET patients and 289 controls	Lack of association with ET	[141]
Singapore	*Leucine-rich repeat kinase 2* (*LRRK2*, *dardarin*, *PARK8*)	12q12	609007/120892	R1628P (rs33949390) variant	450 ET patients and 827 controls	Association of the R1628P variant with the risk for ET, with an OR (95% CI) = 2.20 (1.30–3.73)	[142]
Singapore	*Leucine-rich repeat kinase 2* (*LRRK2*, *dardarin*, *PARK8*)	12q12	609007/120892	R1398 H (rs7133914) and N551K (rs7308720) variants	518 ET patients and 2680 controls	Non-significant trend towards association with the risk for ET. OR (95% CI) = 0.71–1.17 for R1398H, and 0.89 (0.69–1.15) for N551K	[143]
China	*Leucine-rich repeat kinase 2* (*LRRK2*, *dardarin*, *PARK8*)	12q12	609007/120892	rs34594498, rs34410987, and rs33949390	200 ET patients and 434 controls (Chinese Han)	Lack of association with ET	[144]
China	*Leucine-rich repeat kinase 1 gene* (*LRRK1*)	15q26.3	610986/79705	rs2924835	200 ET patients and 434 controls (Chinese Han)	Lack of association with ET	[144]
Spain	*CYP2C9* *CYP2C8*	10.q24 10q23.3	601130/− 601129/−	CYP2C9*2 and *3 CYP2C8 *3	200 ET patients and 300 controls	1.6-fold reduction in the frequency for CYP2C8*3 (*p* = 0.006), 1.35-fold reduction of CYP2C9*2 (*p* = 0.05). 1.52-fold reduction in the frequency for CYP2C9*3 (*p* = 0.07), and 1.33 fold reduction of frequency of at least one defective allele in ET patients (*p* = 0.002). Reduction in the percentage for carriers of the haplotype CYP2C8*3 * CYP2C9*2 in ET patients, *p* = 0.0001 as compared to controls.	[145]
Spain	*Alcohol dehydrogenase 1B* (*ADH1B*)	4q23	103720/125	ADH2 *2 (rs1229984)	204 ET patients and 200 controls	Lack of association with ET	[146]
China	*Alcohol dehydrogenase 1B* (*ADH1B*)	4q23	103720/125	rs6413413 rs1229984	200 ET patients and 229 controls	Lack of association with ET	[147]
Spain	*Glutathione transferase Pi 1* (*GSTP1*)	11q13.2	134660/2950	*GSTP1* Ile105Val (rs1695)	200 ET patients and 220 controls	Lack of association with ET, with the exception of a significantly higher frequency of mutated allelic variants in ET patients exposed to pesticides than in non-exposed.	[148]
Spain	*Histamine-N-methyl-transferase* (*HNMT*)	2q22.1	605238/3176	*HNMT* Thr105Ile (rs11558538)	204 ET patients and 295 controls	Association between homozygous HNMT rs511558538 genotypes leading to high metabolic activity (*p* < 0.015), and the risk for ET (specially for late-onset ET)	[149]
United States of America	*Histamine-N-methyl-transferase* (*HNMT*)	2q22.1	605238/3176	*HNMT* Thr105Ile (rs11558538)	338 ET patients and 409 controls	Lack of association with ET	[150]
Spain	*Paraoxonase 1* (*PON1*)	7q21.3	168820/5444	*PON1* Leu55Met (rs854560) *PON1* Gln192Arg (rs662)	201 ET patients and 220 controls	Lack of association with ET	[151]
United States of America	*Glucosylceranidase beta* (*GBA*)	1q22	606463/2629	*GBA* gene mutations	93 ET patients and 62 controls (all Ashkenazi Jewish)	GBA mutations present in 7.5% (7/93) of ET patients and cases and 4.8% (3/62) of controls. Identification of 4 different heterozygous mutations (3 previously reported mutations— N370S, R496H, E326K—and 1 new missense variant—R44C).	[141]
China	*Glucocerebrosidase* (*GBA*)	1q22	606463/2629	L444P mutation	109 ET patients and 657 controls	Lack of association with ET (0 ET patients and 1 control have heterozygote mutation)	[152]
United States of America	*Microtubule-associated protein tau* (*MAPT*)	17q21.31	157140/4137	rs1052553rs242557	356 ET patients and 409 controls	Association between rs1052553G allele and risk for ET with an OR (95% CI) = 1.32 (1.03–1.67) Lack of association between rs242557 and risk for ET	[153]
Spain	*Microtubule-associated protein tau* (*MAPT*)	17q21.31	157140/4137	rs1052553	200 ET patients and 291 controls	Lack of association with ET in this study and in the pooled data with those of another study [153]	[154]
Spain	*Microtubule-associated protein tau* (*MAPT*)	17q21.31	157140/4137	rs1052553	45 ET patients and 13 subjects without tremor from 11 families with ET and 308 controls	Increased frequency of rs1052553AA genotype and rs1052553A allele in ET patients compared with controls, but lack of association of this allele with the risk for ET in family-based association test	[52]
Canada and United States of America	*Vacuolar protein sorting 35 homolog retromer complex component* (*VPS35*, *PARK17*)	16q11.2	601501/55737	c.1858G > A (rs188286943)	571 ET patients	Presence of the variant studied in 2 of 571 patients	[155]
Canada and United States of America	*DnaJ* (*Hsp40*) *homolog*, *subfamily C*, *member 13* (*DNAJC13*, *PARK21*)	3q22.1	614334/23317	Asn855Ser (rs387907571)	571 ET patients	Lack of association with ET	[155]
Spain, Italy, Germany, North America, and Taiwan	*Triggering receptor expressed on myeloide cells 2* (*TREM2*)	6p21.1	605086/54209	Arg47Leu (rs75932628)	1753 ET patients and 4164 controls (456 ET/2715 controls from Spain; 897 ET/1449 controls from other populations)	Increased risk for ET in carriers of the variant in the Spanish cohort, with an OR (95% CI) = 5.97 (1.203–29.626), but lack of association in the cohort of other populations	[156]
Spain	*Heme-oxygenase 1* (*HMOX1*)	22q12.3	141250/3162	rs2071746 rs2071747 Copy number variations (CNV)	202 patients with familial ET and 747 controls	Decreased risk for ET in carriers of rs2071746T allele	[157]
China	*Heme-oxygenase 1* (*HMOX1*)	22q12.3	141250/3162	rs2071746	200 ET patients and 229 controls	Lack of association with ET	[147]
Spain	*Heme-oxygenase 2* (*HMOX2*)	16p13.3	141251/3163	rs2270363 rs1051308 Copy number variations (CNV)	202 patients with familial ET, and 747 controls	Decreased risk for ET in carriers of rs1051308G allele	[157]
China	*Heme-oxygenase 2* (*HMOX2*)	16p13.3	141251/3163	rs4786504 rs1051308	200 ET patients and 229 controls	Lack of association with ET	[147]
Iran	*Ras like without CAAX 2* (*RIT2*)	18q12.3	609592/6014	rs12456492 rs16976358	350 ET patients and 1000 controls	Association between rs12456492 and risk for ET in additive and in recessive models, with OR (95% CI) 1.37 (1.11–1.70) and 2.21 (1.47–3.30), respectively	[158]
China	*Fibroblast growth factor 20* (*FGF20*)	8p22	605558/26281	rs1721100 rs1989754 rs10089600 rs12720208 rs17550360	200 ET patients and 426 controls (Chinese Han)	Lack of association with ET	[159]
China	*Paired-like homeo-domain 3* (*PITX3*)	10q24.32	602669/5309	rs3758549 rs4919621	200 ET patients and 426 controls (Chinese Han)	Lack of association with ET	[160]
China	*C*oiled-coil-helix-coiled-coil-helix domain containing 2 (*CHCHD2*, *PARK22*)	7p11.22	616244/51142	182C>T (Thr61Ile)	171 familial ET patients and 211 controls (Chinese Han)	Lack of detection of mutations in patients and controls	[161]
China	*Transmembrane protein 230* (*TMEM230*)	20p13-p12.3	617019/29058	Stop codon TAG	200 ET patients (100 with positive family history) and 400 controls	Lack of detection of mutations in patients and controls	[162]
Turkey	*Vitamin D3 receptor* (*VDR*)	12q.13.11	601769/7421	rs2228570	239 ET patients and 239 controls	Increased risk for ET in carriers of the rs2228570C (major) allele with an OR (95% CI) = 2.207 (1.051–4.636)	[163]
China	*Vitamin D3 receptor* (*VDR*)	12q.13.11	601769/7421	rs731236	200 ET patients and 229 controls	Lack of association with ET	[147]
China	*Interleukin 17 alpha* (*IL17A*)	6p12.2	603149/3605	rs8193036	200 ET patients and 229 controls	Lack of association with ET	[147]
China	*Interleukin 1-beta* (*IL1B*)	2q14.1	147720/3553	rs1143633, rs1143643, rs1143634	200 ET patients and 229 controls	Association between rs1143633 allele and the risk for ET, with an OR (95% CI) = 2.57 (1.38–4.81)	[147]
China	*Nitric oxide synthase 1* (*NOS1*)	12q24.22	163731/4842	rs693534, rs7977109	200 ET patients and 229 controls	Lack of association with ET	[147]
China	*NUS1 dehydrodoli-chyl diphosphate synthase subunit* (*NUS1*)	6q22.1	610463/116150	Sequencing of the 5 coding regions and the exon-intron boundaries of the gene	395 ET patients and 395 controls	Lack of association with ET	[164]

OR: odds ratio (OR); 95% CI: 95% confidence intervals (CI); NAD: non-available data.

**Table 3:** Data from other studies of possible candidate genes for ET.

Country	Gene	Chromosome	MIM/ Gene Id	Allelic Variant/ Mutations	Rationale	Study Design or Participants	Main Results	[Ref]
France	*Torsin* family 1 member A *1A*, *Tor1A* or *DYT1*	9q34.11	605204/1861	Mutations in *DYT1* gene	Postural tremor is a frequent clinical feature of idiopathic torsion dystonia	Linkage analysis for locus *DYT1* in two large families with ET	Lack of association with ET	[165]
United Kingdom	*Torsin* family 1 member A *1A*, *Tor1A* or *DYT1*	9q34.11	605204/1861	Mutations in *DYT1* gene at the argininosuccinate-synthase (ASS) and Abelson loci	Postural tremor is a frequent clinical feature of idiopathic torsion dystonia	Linkage analysis for locus *DYT1* in 15 large families with ET (60 affected individuals)	Lack of association with ET	[166]
Russia	*Torsin* family 1 member A *1A*, *Tor1A* or *DYT1*	9q34.11	605204/1861	Mutations in *DYT1* gene	Postural tremor is a frequent clinical feature of idiopathic torsion dystonia	Linkage analysis for locus *DYT1* in a group of Slavonic (11 patients) and Tajik (19 patients) families with ET.	Lack of association with ET	[34]
Italy	*Potasium Channel*, *Calcium-activate*, *intermediate*/*small conductance*, *subfamily N*, *member 3* (*KCCN3* or *SKCA3*)	1q21.3	602983/3782	CAG repeat expansions	Some studies linked this gene with the risk for schizophrenia (not confirmed in others) and with juvenile myoclonic epilepsy	88 ET patients (78 familial ET) and 78 controls	Lack of association with ET	[167]
Italy	*Calcium Channel*, *Voltage-dependent*, *P*/*Q type*, *Alpha-1A subunit* (*CACNA1A*, *CACNA1A4*, or *SCA6)*	19p13.13	601011/773	CAG repeat expansions	Relation with episodic ataxia, type 2, migraine familial hemiplegic, and spinocerebellar ataxia 6	98 ET patients (88 familial ET) and 94 controls	Lack of association with ET	[167]
United States of America	*Calcium Channel*, *Voltage-dependent*, *P*/*Q type*, *Alpha-1A subunit* (*CACNA1A*, *CACNA1A4*, or *SCA6)*	19p13.13	601011/773	Pathogenic repeat expansions	SCA3 and other SCAs can present initially with ET symptoms	323 ET patients and 299 controls	Lack of association with ET	[168]
Italy	*Spinocerebellar ataxia 12* (*SCA12*)	5q31-q33	604326/5521	CAG repeat expansions	Action tremor of the head and arms is very often present in early stages of SCA12	30 ET patients	None of 30 ET patients presented a CAG repeat larger than 19	[169]
United States of America	*Spinocerebellar ataxia 12* (*SCA12*)	5q31-q33	604326/5521	CAG repeat expansions	SCA3 and other SCAs can present initially with ET symptoms	323 ET patients and 299 controls	Lack of association with ET	[168]
Singapore	*Ataxia 3*, *spinocerebellar ataxia 3* (*ATXN3*, *SCA3*)	14q32.12	607047/4287	Pathogenic repeat expansions	SCA3 and other SCAs can present initially with ET symptoms	177 ET patients	*SCA3* mutations were present in 1 of 177 ET patients	[170]
United States of America	*Ataxin 3*, *spinocerebellar ataxia 3* (*ATXN3*, *SCA3*)	14q32.12	607047/4287	Pathogenic repeat expansions	SCA3 and other SCAs can present initially with ET symptoms	323 ET patients and 299 controls	Lack of association with ET	[168]
Singapore	*Ataxin 2*, *spinocerebellar ataxia 2* (*ATX1*, *SCA2*)	12q24.12	601517/6311	Pathogenic repeat expansions	SCA3 and other SCAs can present initially with ET symptoms	177 ET patients	None of the 177 ET patients presented with *SCA3* mutations	[170]
United States of America	*Ataxin 2*, *spinocerebellar ataxia 2* (*ATX1*, *SCA2*)	12q24.12	601517/6311	Pathogenic repeat expansions	SCA3 and other SCAs can present initially with ET symptoms	323 ET patients and 299 controls	Significant differences in the distribution of repeats in the ‘normal’ range for SCA2 between ET patients and controls.	[168]
United States of America	*Ataxin 1*, *spinocerebellar ataxia 1* (*ATXN1*, *SCA1*)	6p22.3	601556/6310	Pathogenic repeat expansions	SCA3 and other SCAs can present initially with ET symptoms	323 ET patients and 299 controls	Lack of association with ET	[168]
United States of America	*Ataxin 7*, *spinocerebellar ataxia 7* (*ATXN7*, *SCA7*)	3p14.1	607640/6314	Pathogenic repeat expansions	SCA3 and other SCAs can present initially with ET symptoms	323 ET patients and 299 controls	Lack of association with ET	[168]
United States of America	*Ataxin 8*, *spinocerebellar ataxia 8* (*ATXN8*, *SCA8*)	13q21	613289/724066	Pathogenic repeat expansions	SCA3 and other SCAs can present initially with ET symptoms	323 ET patients and 299 controls	Significant differences in the distribution of repeats in the ‘normal’ range for SCA8 between ET patients and controls.	[168]
United States of America	*Ataxin 10*, *spinocerebellar ataxia 10* (*ATXN8*, *SCA10*)	22q13.31	611150/25814	Pathogenic repeat expansions	SCA3 and other SCAs can present initially with ET symptoms	323 ET patients and 299 controls	Lack of association with ET	[168]
United States of America	*TATA-box binding protein* (*TBP*, *SCA17*)	6q27	600075/6908	Pathogenic repeat expansions	SCA3 and other SCAs can present initially with ET symptoms	323 ET patients and 299 controls	Lack of association with ET	[168]
United States of America	*Atrophin 1*, *dentatorubral-pallidolysian atrophy* (*ATN1*, *DRPLA*)	12p13.31	607432/1822	Pathogenic repeat expansions	SCA3 and other SCAs can present initially with ET symptoms	323 ET patients and 299 controls	Lack of association with ET	[168]
United States of America and Canada	*FMRP translational regulator 1* (*FMR1)*, *fragile X-associated tremor*/*ataxia syndrome* (*FXTAS*)	Xq27.3	309550/2332	Pathogenic mutations in *FMR1*	FXTAS can present with ET phenotype	2 ET patients with *FMR1* mutations	Description of 2 patients with FMR1 and ET phenotype from two large University Movement Disorders Clinics	[171]
Singapore	*FMRP translational regulator 1* (*FMR1)*, *fragile X-associated tremor*/*ataxia syndrome* (*FXTAS*)	Xq27.3	309550/2332	Pathogenic muta-tions in *FMR1* (premutations alleles, 55–200 CGG repeats)	FXTAS can present with ET phenotype	71 ET patients and 200 controls	None of the ET patients or controls carried alleles within the premutation range	[172]
United States of America	*FMRP translational regulator 1* (*FMR1*), *fragile X-associated tremor*/*ataxia syndrome* (*FXTAS*)	Xq27.3	309550/2332	Pathogenic muta-tions in *FMR1* (premutations alleles, 55–200 CGG repeats)	FXTAS can present with ET phenotype	81 ET patients	None of the ET patients carried alleles within the premutation range	[173]
United States of America	*FMRP translational regulator 1* (*FMR1*), *fragile X-associated tremor*/*ataxia syndrome* (*FXTAS*)	Xq27.3	309550/2332	Pathogenic muta-tions in *FMR1* (premutations alleles, 55–200 CGG repeats)	FXTAS can present with ET phenotype	196 ET male patients	None of the ET patients carried alleles within the premutation range	[174]
United Kingdom	*FMRP translational regulator 1* (*FMR1*), *fragile X-associated tremor*/*ataxia syndrome* (*FXTAS*)	Xq27.3	309550/2332	Pathogenic mutations in *FMR1*	FXTAS can present with ET phenotype	1 ET patients with *FMR1* mutations	Description of 1 patient with FMR1 and ET phenotype	[175]
United States of America	*FMRP translational regulator 1* (*FMR1*), *fragile X-associated tremor*/*ataxia syndrome* (*FXTAS*)	Xq27.3	309550/2332	Pathogenic muta-tions in *FMR1* (premutations alleles, 55–200 CGG repeats)	FXTAS can present with ET phenotype	321 ET patients and 296 controls	None of the ET patients or controls carried alleles within the premutation range or in “grey zone” (41–54 CGG repeats)	[176]
South Korea	*FMRP translational regulator 1* (*FMR1*), *fragile X-associated tremor*/*ataxia syndrome* (*FXTAS*)	Xq27.3	309550/2332	Pathogenic muta-tions in *FMR1* (premutations alleles, 55–200 CGG repeats)	FXTAS can present with ET phenotype	74 patients with ET and cerebellar and/or extrapyramidal signs selected from a cohort of 603 patients diagnosed with ET	Two of these 74 patients (2.7%) had a FMR1 premutation and fulfilled both clinical and radiological criteria of FXTAS	[177]
United Status of America	*Amino-levulinic acid dehydratase* (*ALAD*)	9q33.1	125270/210	rs1800435	Role of ALAD in the synthesis of hemoproteins, being inhibited by lead. Increased serum lead levels are associated with risk for ET	63 ET patients and 101 controls	Lack of direct association with the risk for ET, but increased OR for ET in patients with high lead levels carrying the minor allele	[178]
Spain	*Amino-levulinic acid dehydratase* (*ALAD*)	9q33.1	125270/210	rs1800435	Role of ALAD in the synthesis of hemoproteins, being inhibited by lead. Increased serum lead levels are associated with risk for ET.	202 ET patients and 218 controls	Lack of direct association with the risk for ET. Interaction between rs1800435CC genotype (wild-type) with *HMOX2* rs1051308GG genotype or G allele decreased risk for ET.	[179]
South Korea	Mitochondrial genes	-	-	Mitochondrial DNA (mtDNA)	Association of alterations in mitochondrial genes with some neurodegenerative diseases.	9 familial ET patients and 6 controls	Several deletions identified in a small group of patients with TE. These affect areas in complexes I, III, IV, and V.	[180]
United States of America	*Sodium*, *Voltage-gated Channel alpha subunit type 8* (*SNC8A* or *NAV1.6*)	12q13.13	600702/6334	Mutations in *SNC8A* gene	Mutations in this gene cause congenital tremor in mice.	95 patients with familial ET (48 of them with early onset)	Lack of association with ET	[181]
Spain	*Gamma-aminobutyric acid type A receptor subunit rho1 GABR Rho1* (*GABRR1*)	6q15	137161/2569	*GABRR1 26 V* (rs12200969) *GABRR1 27R* (rs1186902)	*Knockout GABRA1* mice show postural and kinetic tremor and motor incoordination resembling human ET	200 ET patients and 250 controls	Lack of association with ET	[182]
Spain	*Gamma-aminobutyric acid type A receptor subunit rho2 GABR Rho2* (*GABRR2*)	6q15	137162/2570	*GABRR2 455M* (rs282129)	*Knockout GABRA1* mice show postural and kinetic tremor and motor incoordination resembling human ET	200 ET patients and 250 controls	Lack of association with ET	[182]
Spain	*Gamma-aminobutyric acid type A receptor subunit rho3 GABR Rho3* (*GABRR3*)	3q11.2	618668/200959	*GABRR3 205Y* (rs832032)	*Knockout GABRA1* mice show postural and kinetic tremor and motor incoordination resembling human ET	200 ET patients and 250 controls	Lack of association with ET	[183]
Spain	* Gamma-aminobutyric acid type A receptor subunit alpha4 GABR Alpha4* (*GABRA4*)	4p12	137141/2557	*GABRA4 26M* (rs2229940)	*Knockout GABRA1* mice show postural and kinetic tremor and motor incoordination resembling human ET	200 ET patients and 250 controls	Lack of association with ET	[183]
Spain	*Gamma-aminobutyric acid type A receptor subunit epsilon GABR Epsilon* (*GABRE*)	Xp28	300093/2564	*GABRE 102S* (rs1139916)	*Knockout GABRA1* mice show postural and kinetic tremor and motor incoordination resembling human ET	200 ET patients and 250 controls	Lack of association with ET	[183]
Spain	*Gamma-aminobutyric acid type A receptor subunit theta GABR Theta* (*GABRQ*)	Xq28	300349/55879	*GABRQ 4478F* (rs3810651)	*Knockout GABRA1* mice show postural and kinetic tremor and motor incoordination resembling human ET	200 ET patients and 250 controls	Lack of association with ET	[183]
Germany and Denmark	192 tagging SNPs (tagSNP) for 14 GABA-A receptor genes and 48 tagSNP for 4 GABA transporter genes (the genes and SNPs specified below were associated with ET in the first analysis)	-	-	-	*Knockout GABRA1* mice show postural and kinetic tremor and motor incoordination resembling human ET. *Knockout* mice for the *GABA transporter* (*GAT*) *GAT-1* gene show a motor disorder, including a 25 to 32 Hz tremor	503 ET patients and 818 controls	Lack of association with ET	[184]
Germany and Denmark	*Gamma-aminobutyric acid type A receptor subunit gamma1* (*GABRG1*)	4p12	137166/2565	rs6833256	*Knockout GABRA1* mice show postural and kinetic tremor and motor incoordination resembling human ET. *Knockout* mice for the *GABA transporter* (*GAT*) *GAT-1* gene show a motor disorder, including a 25 to 32 Hz tremor	503 ET patients and 818 controls	Lack of association with ET	[184]
Germany and Denmark	*Gamma-aminobutyric acid type A receptor subunit beta1* (*GABRB1*)	4p12	137190/2560	rs971353	*Knockout GABRA1* mice show postural and kinetic tremor and motor incoordination resembling human ET. *Knockout* mice for the *GABA transporter* (*GAT*) *GAT-1* gene show a motor disorder, including a 25 to 32 Hz tremor	503 ET patients and 818 controls	Lack of association with ET	[184]
Germany and Denmark	*Gamma-aminobutyric acid type A receptor subunit pi* (*GABRP*)	5q35.1	602729/2568	rs1559159 rs11745599 rs7722089	*Knockout GABRA1* mice show postural and kinetic tremor and motor incoordination resembling human ET. *Knockout* mice for the *GABA transporter* (*GAT*) *GAT-1* gene show a motor disorder, including a 25 to 32 Hz tremor	503 ET patients and 818 controls	Lack of association with ET	[184]
Germany and Denmark	*Gamma-aminobutyric acid type A receptor subunit beta3* (*GABRB3*)	15q12	137192/2562	rs4542636	*Knockout GABRA1* mice show postural and kinetic tremor and motor incoordination resembling human ET. *Knockout* mice for the *GABA transporter* (*GAT*) *GAT-1* gene show a motor disorder, including a 25 to 32 Hz tremor	503 ET patients and 818 controls	Lack of association with ET	[184]
Germany and Denmark	*Gamma-aminobutyric acid type A receptor subunit gamma3* (*GABRG3*)	15q12	600233/2567	rs11635966 rs6606877 rs4887564	*Knockout GABRA1* mice show postural and kinetic tremor and motor incoordination resembling human ET. *Knockout* mice for the *GABA transporter* (*GAT*) *GAT-1* gene show a motor disorder, including a 25 to 32 Hz tremor	503 ET patients and 818 controls	Lack of association with ET	[184]
China	*Meis homeobox 1* (*MEIS1*)	2p14	601739/4211	rs4544423 rs6710341 rs12469063 rs2300478	Relationship between ET and restless legs syndrome (RLS)	200 ET patients and 201 controls	Lack of association with ET	[185]
China	*BTB domain containing 9* (*BTBD9*)	6p21.2	611237/114781	rs9296249 rs9357271 rs3923809	Relationship between ET and restless legs syndrome (RLS)	200 ET patients and 201 controls	Lack of association with ET	[185]
China	*Protein tyrosine phosphatase receptor type D* (*PTPRD*)	9p24.1-p23	601598/5789	rs10977209 rs1975197 rs4626664	Relationship between ET and restless legs syndrome (RLS)	200 ET patients and 201 controls	Lack of association with ET	[185]
China	*Mitogen-activated protein kinase 5* (*MAP2K5*) *SKI family transcriptional corepressor 1* (*SKOR1*, *LBXCOR1*)	15q23	602520/5607 611273/390590	rs12593813 rs11635424 rs4489954 rs3784709 rs2241420 rs1026732 rs6494696	Relationship between ET and restless legs syndrome (RLS)	200 ET patients and 201 controls	rs6494696 SNP and haplotype (rs4489954, rs3784709, rs2241420, rs1026732, and rs6494696) were associated with the risk for ET	[185]
China	*TOX high mobility group box family member 3* (*TOX3*)	16q12.1	611416/27324	rs3104767 * rs3104788 *	Relationship between ET and restless legs syndrome (RLS)	200 ET patients and 201 controls	rs3104767 SNP showed association with ET risk	[185]
China	Intergenic region	2p14	-	rs6747972	Relationship between ET and restless legs syndrome (RLS)	200 ET patients and 201 controls	rs6747972 SNP showed association with ET risk	[185]
Canada	*Meis homeobox 1* (*MEIS1*)	2p14	601739/4211	rs4544423 rs6710341 rs12469063 rs2300478	Relationship between ET and restless legs syndrome (RLS)	1778 ET patients and 5376 controls	Lack of association with ET	[186]
Canada	*BTB domain containing 9* (*BTBD9*)	6p21.2	611237/114781	rs9296249 rs9357271 rs3923809	Relationship between ET and restless legs syndrome (RLS)	1778 ET patients and 5376 controls	Lack of association with ET	[186]
Canada	*Protein tyrosine phosphatase receptor type D* (*PTPRD*)	9p24.1-p23	601598/5789	rs10977209 rs1975197 rs4626664	Relationship between ET and restless legs syndrome (RLS)	1778 ET patients and 5376 controls	Lack of association with ET	[186]
Canada	*Mitogen-activated protein kinase 5* (*MAP2K5*) *SKI family transcriptional corepressor 1* (*SKOR1*, *LBXCOR1*)	15q23	602520/5607611273/390590	rs12593813 rs11635424 rs4489954 rs3784709 rs2241420 rs1026732 rs6494696	Relationship between ET and restless legs syndrome (RLS)	1778 ET patients and 5376 controls	Lack of association with ET	[186]
Canada	*TOX high-mobility group box family member 3* (*TOX3*)	16q12.1	611416/27324	rs3104767 * rs3104788 *	Relationship between ET and restless legs syndrome (RLS)	1778 ET patients and 5376 controls	Lack of association with ET	[186]
Canada	Intergenic region	2p14	-	rs6747972	Relationship between ET and restless legs syndrome (RLS)	1778 ET patients and 5376 controls	Lack of association with ET	[186]
Canada	*Calcium voltage-gated channel subunit alpha1 C* (*CACNA1C*)	12p13.33	114205/775	9 variants, 3 missense	Gene enriched for their expression in the olivocerebellar motor circuitry; calcium and glutamate signaling pathways	262 ET patients and 283 controls	Lack of association with ET	[186]
Canada	*Calcium voltage-gated channel subunit alpha1 E* (*CACNA1E*)	1q25.3	601013/777	19 variants, 9 missense	Gene enriched for their expression in the olivocerebellar motor circuitry; calcium and glutamate signaling pathways	262 ET patients and 283 controls	Lack of association with ET	[187]
Canada	*Calcium voltage-gated channel subunit alpha1 G* (*CACNA1G*)	17q21.33	604065/8913	8 variants, 4 missense	Gene enriched for their expression in the olivocerebellar motor circuitry; calcium and glutamate signaling pathways	262 ET patients and 283 controls	Lack of association with ET	[187]
Canada	*Calcium voltage-gated channel auxiliary subunit beta 3* (*CACNB3*)	12q13.12	601958/784	1 variants, 0 missense	Gene enriched for their expression in the olivocerebellar motor circuitry; calcium and glutamate signaling pathways	262 ET patients and 283 controls	Lack of association with ET	[187]
Canada	*Calmodulin 3* (*CALM3*)	19q13.32	114183/808	Not specified, 0 missense	Gene enriched for their expression in the olivocerebellar motor circuitry; calcium and glutamate signaling pathways	262 ET patients and 283 controls	Lack of association with ET	[187]
Canada	*Calcium/calmodulin-dependent protein kinase II alpha* (*CAMK2A*)	5q32	114078/815	4 variants, 1 missense	Gene enriched for their expression in the olivocerebellar motor circuitry; calcium and glutamate signaling pathways	262 ET patients and 283 controls	Lack of association with ET	[187]
Canada	*Glutamate ionotropic receptor NMDA type subunit 3A* (*GRIN3A*)	9q31.1	606650/116443	16 variants, 11 missense	Gene enriched for their expression in the olivocerebellar motor circuitry; calcium and glutamate signaling pathways	262 ET patients and 283 controls	Lack of association with ET	[187]
Canada	*Glutamate metabotropic receptor 5* (*GRM5*)	11q14.2-q14.3	604102/2915	3 variants, 0 missense	Gene enriched for their expression in the olivocerebellar motor circuitry; calcium and glutamate signaling pathways	262 ET patients and 283 controls	Lack of association with ET	[187]
Canada	*5-hydroxytryptamine receptor 2C* (*HTR2C*)	Xq23	312861/3358	1 variants, 1 missense	Gene enriched for their expression in the olivocerebellar motor circuitry; calcium and glutamate signaling pathways	262 ET patients and 283 controls	Lack of association with ET	[187]
Canada	*Solute carrier family 17 member 6* (*SLC17A6*)	11p14.3	607563/57084	7 variants, 3 missense	Gene enriched for their expression in the olivocerebellar motor circuitry; calcium and glutamate signaling pathways	262 ET patients and 283 controls	Lack of association with ET	[187]
Canada	*Solute carrier family 1 member 1* (*SLC1A1*)	9p24.2	133550/6505	6 variants, 2 missense	Gene enriched for their expression in the olivocerebellar motor circuitry; calcium and glutamate signaling pathways	262 ET patients and 283 controls	Lack of association with ET	[187]

* According to the dbSNP database, rs3104767 and rs3104788 SNPs belong to the *cancer susceptibility 16* (*CASC16*) gene (chromosome 16q12.1-q12.2; MIM not available; Gene ID 643714).

**Table 4:** Design recommendations for studies focused on genetic research of essential tremor (adapted from text of Reference [23]).

Selection of Index Patients and Controls
Index patients should have a positive family history of ET and be diagnosed with definite and ‘‘pure’’ or “monosymptomatic” ET according to standardized criteria.
Index patients could participate both in family studies and in case-control association studies or family studies.
Inclusion of controls in case-control association studies as “healthy” should imply the absence of a family history of tremor and other movement disorders and the neurological interview and examination to exclude the presence of tremor or other movement disorders.
**Selection of Relatives**
All available first-degree relatives of the index patient should undergo a clinical examination, including rating scales for tremor.
ET families should be divided into several subtypes, that should be sub-analyzed separately, according to the coexistence or not of other neurological diseases such as dystonia and PD (“pure ET”, “ET-dystonia”, “ET-PD”, “ET-dystonia”).
**Study Design**
Multicenter, multiethnic, and prospective design.
Long-term follow-up to assess further development of PD or other associated disorders in the index patients, in their relatives, or both, and development of ET during the follow-up period by relatives of ET patients who had no tremor in the initial assessment
**Blood Collection**
Obtention of blood for DNA extraction both from patients, their relatives, and healthy controls. The samples obtained will be used for future genetic studies attempting to establish the role of genetic factors in the different clinical subtypes of ET.

## Data Availability

No new data were created or analyzed in this study. Data sharing is not applicable to this article.
